# Air Pollution Measurement and Dispersion Simulation Using Remote and In Situ Monitoring Technologies in an Industrial Complex in Busan, South Korea

**DOI:** 10.3390/s24237836

**Published:** 2024-12-07

**Authors:** Naghmeh Dehkhoda, Juhyeon Sim, Juseon Shin, Sohee Joo, Sung Hwan Cho, Jeong Hun Kim, Youngmin Noh

**Affiliations:** 1 Division of Earth Environmental System Science, Pukyong National University, Busan 48513, Republic of Korea; melody87@pukyong.ac.kr (N.D.); sjh10120901@pukyong.ac.kr (J.S.); juseonshin@pukyong.ac.kr (J.S.); thgml1gh@pukyong.ac.kr (S.J.); 2 Air Pollution Engineering Division, National Institute of Environmental Research, Incheon 22689, Republic of Korea; joshwan@korea.kr (S.H.C.); magnus@korea.kr (J.H.K.); 3 Department of Environmental Engineering, Pukyong National University, Busan 48513, Republic of Korea

**Keywords:** solar occultation flux (SOF), sky differential optical absorption spectroscopy (SkyDOAS), mobile extraction Fourier transform infrared spectrometry (MeFTIR), total alkanes, sulfur dioxide (SO_2_), nitrogen dioxide (NO_2_), formaldehyde (HCHO), methane (CH_4_), hybrid single-particle Lagrangian integrated trajectory (HYSPLIT) diffusion model

## Abstract

Rapid industrialization and the influx of human resources have led to the establishment of industrial complexes near urban areas, exposing residents to various air pollutants. This has led to a decline in air quality, impacting neighboring residential areas adversely, which highlights the urgent need to monitor air pollution in these areas. Recent advancements in technology, such as Solar Occultation Flux (SOF) and Sky Differential Optical Absorption Spectroscopy (SkyDOAS) used as remote sensing techniques and mobile extraction Fourier Transform Infrared Spectrometry (MeFTIR) used as an in situ technique, now offer enhanced precision in estimating the pollutant emission flux and identifying primary sources. In a comprehensive study conducted in 2020 in the Sinpyeong Jangrim Industrial Complex in Busan City, South Korea, a mobile laboratory equipped with SOF, SkyDOAS, and MeFTIR technologies was employed to approximate the emission flux of total alkanes, sulfur dioxide (SO_2_), nitrogen dioxide (NO_2_), formaldehyde (HCHO), and methane (CH_4_). Using the HYbrid Single-Particle Lagrangian Integrated Trajectory (HYSPLIT) diffusion model, pollutant dispersion to residential areas was simulated. The highest average daily emission flux was observed for total alkanes, with values of 69.9 ± 71.6 kg/h and 84.1 ± 85.8 kg/h in zones S_1_ and S_2_ of the Sinpyeong Jangrim Industrial Complex, respectively. This is primarily due to the prevalence of metal manufacturing and mechanical equipment industries in the area. The HYSPLIT diffusion model confirmed elevated pollution levels in residential areas located southeast of the industrial complex, underscoring the influence of the dominant northwesterly wind direction and wind speed on pollutant dispersion. This highlights the urgent need for targeted interventions to address and mitigate air pollution in downwind residential areas. The total annual emission fluxes were estimated at 399,984 kg/yr and 398,944 kg/yr for zones S_1_ and S_2_, respectively. A comparison with the Pollutant Release and Transfer Registers (PRTRs) survey system revealed that the total annual emission fluxes in this study were approximately 24.3 and 4.9 times higher than those reported by PRTRs. This indicates a significant underestimation of the impact of small businesses on local air quality, which was not accounted for in the PRTR survey system.

## 1. Introduction

The surge in factory-based production, economic growth, and rapid industrialization has significantly escalated air pollutant emissions from industrial regions. These pollutants, including nitrogen oxides (NO_X_), sulfur oxides (SO_X_), volatile organic compounds (VOCs), alkanes, alkenes, and formaldehyde (HCHO) not only pose risks to human health but also contribute to the degradation of air quality [1]. Among the monitored anthropogenic VOCs, benzene, toluene, ethylbenzene, and xylene (BTEX) feature prominently, primarily from motor vehicle emissions [2]. Tropospheric ozone formation arises from the chemical interplay between NO_X_ and VOCs, catalyzed by sunlight exposure [3]. This happens when pollutants emitted by cars, power plants, industrial boilers, refineries, chemical plants, and other sources chemically react in sunlight.

The concentration of anthropogenic sources in urban areas often challenges cities to meet air quality standards. Busan, South Korea’s second most populous city after Seoul, hosts the Sinpyeong Jangrim Industrial Complex, comprising 497 industrial facilities spanning metal manufacturing, mechanical and electrical equipment, food and beverage industries, textile and clothing production, transportation equipment, automobile manufacturing, rubber and plastic production, wood and paper manufacturing, etc. Consequently, a significant volume of air pollutants, including sulfur dioxide (SO_2_), nitrogen dioxide (NO_2_), methane (CH_4_), alkanes, HCHO, and benzene, is annually released into the atmosphere from these factories. Notably, emissions of alkanes and alkenes from refineries and petrochemical industries primarily stem from evaporative losses from process equipment and storage tanks, while NO_2_ and SO_2_ emissions predominantly emanate from agricultural activities, industrial processes, and external combustion sources. Given that the Sinpyeong Jangrim Industrial Complex is intermixed with residential areas and emits substantial pollutants into the atmosphere, governmental attention has recently shifted towards this complex. In an initial effort, South Korea’s Ministry of Environment’s Chemical Safety Agency created the Pollutant Release and Transfer Registers (PRTRs) survey system to report annual air pollution emissions, including total alkanes, NO_2_, SO_2_, and HCHO, from large industries using optical remote sensing technologies. Although large businesses have a greater impact on atmospheric pollution, ignoring the share of small industries leads to an underestimation of their role in air pollution and regional atmospheric quality.

Modern optical measurement tools like Multi-AXis Differential Optical Absorption Spectroscopy (MAX-DOAS), concurrent DOAS, and scanning Fourier Transform Infrared Spectroscopy (FTIR) are preferred for their capacity to directly measure atmospheric trace gasses without the need for extensive pre- or post-treatment in laboratory settings [4]. The DOAS technique, a remote sensing method for air quality assessment, utilizes scattered sunlight as a light source, scanning it at various elevation angles through sequential scanning with a stepper motor [5]. Meanwhile, FTIR stands as a widely employed and effective technique for capturing an infrared spectrum of absorption or emission from solids, liquids, or gasses, facilitating the quantification of molecules in gas mixtures [6].

Researchers have utilized advanced techniques such as Imaging DOAS (i-DOAS) to discern two-dimensional plume structures from power plant emissions and assess the spatial distribution of NO_2_ and SO_2_ [7]. Concurrent Multi-AXis DOAS (CMAX-DOAS) systems have been developed to measure NO_2_ in urban environments, offering valuable insights into city-wide NO_2_ concentrations [8]. Additionally, the DOAS technique and a mobile platform were employed for flux measurements of alkanes, SO_2_, and NO_2_ emissions using the Solar Occultation Flux (SOF) method in major oil refineries and petrochemical conglomerates in southeast and east Texas [9]. In a separate study, the scanning FTIR method enabled the detection of ammonia (NH_3_) and the visualization of pollutant spatial distribution and concentration through background measurements in agricultural and urban settings [10].

Despite the extensive reach of the Sinpyeong Jangrim Industrial Complex in Busan and the substantial emission of various pollutants from this facility, along with its proximity to residential areas, a comprehensive investigation into pollutant amounts, emission trajectories, and the complex’s environmental footprint has not been conducted yet. Additionally, since the PRTR survey system is only conducted for large businesses, the contribution of small industries to air pollution is ignored. This paper aims to present a detailed analysis of the total alkanes, SO_2_, NO_2_, HCHO, and CH_4_ emission flux utilizing FTIR and DOAS technologies within an industrial complex situated in Busan, South Korea, during 2020. By measuring the emission flux, wind patterns, and the spread of pollutants, this study seeks to identify primary pollution sources and the affected nearby regions. To investigate the contribution of small businesses to air pollution, the results of the PRTR survey system were compared with this study, and the findings are discussed [11]. Moreover, the study employed the HYbrid Single-Particle Lagrangian Integrated Trajectory (HYSPLIT) diffusion model to estimate the dispersion of pollutants into the surrounding areas. Ultimately, this paper discusses the representativeness of its results concerning the prevailing meteorological conditions.

## 2. Material and Methods

### 2.1. Study Area

The Sinpyeong Jangrim Industrial Complex, located 20 km southwest of Busan City in South Korea, serves as a pivotal industrial site encompassing 497 factories. Established between 1981 and 1990 with a 2,602,000 m^2^ area, this complex hosts a diverse array of manufacturing sectors, including metal and non-metal production, mechanical and chemical manufacturing, leatherworks, automobile and transportation equipment fabrication, and textile and clothing industries, as well as plastic and rubber production [12]. The operations of these industries result in the release of various chemical substances into the atmosphere, significantly impacting the surrounding environment.

**Figure 1:**
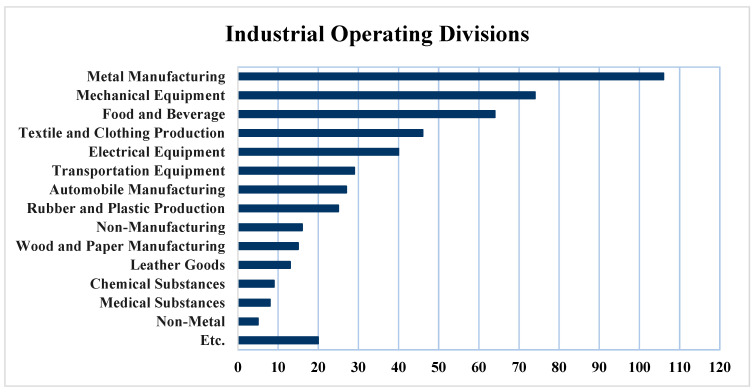
The industrial operating divisions located in the Sinpyeong Jangrim Industrial Complex.

**Figure 2:**
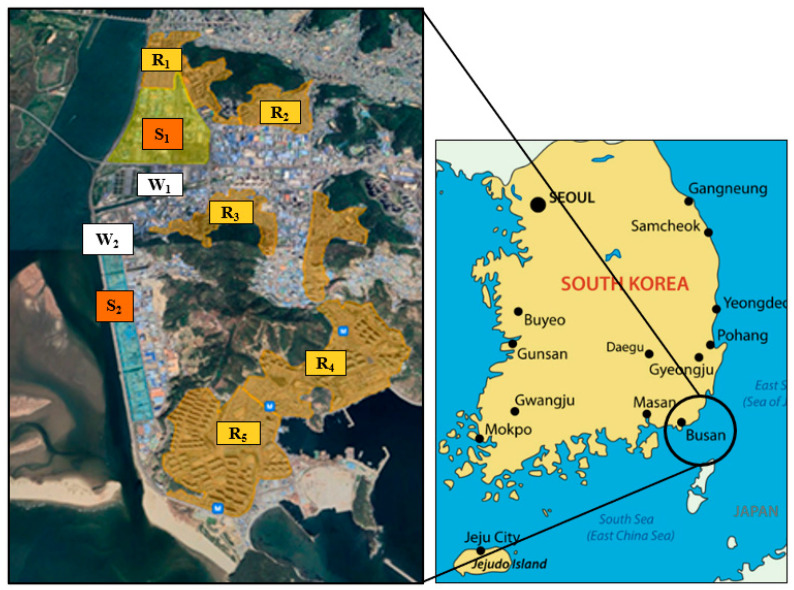
Location of Busan City in South Korea and the Sinpyeong Jangrim Industrial Complex (S_1_ and S_2_), residential areas (R_1_–R_5_), and installed anemometers (W_1_ and W_2_). The purple spots indicate other industrial areas near the observation site.

The Sinpyeong Jangrim Industrial Complex is intermixed with residential areas, and a substantial population comprising 128,548 households is directly exposed to the pollutants emitted by these industrial facilities. Figure 1 introduces the industries located in the Sinpyeong Jangrim Industrial Complex. Figure 2 shows the location of the Sinpyeong Jangrim Industrial Complex (S_1_ and S_2_) and the residential areas (R_1_–R_5_); in addition, the location of two anemometers (to check the wind direction and speed: W_1_ and W_2_) is presented on the map. The study conducted measurements over 8 days in Zone S_1_ and 9 days in Zone S_2_ during November 2020. The strategic focus on this complex underscores its importance as a critical industrial zone in Busan City, substantiating the need for a detailed environmental assessment and ongoing vigilance.

### 2.2. Measurement Techniques

**Figure 3:**
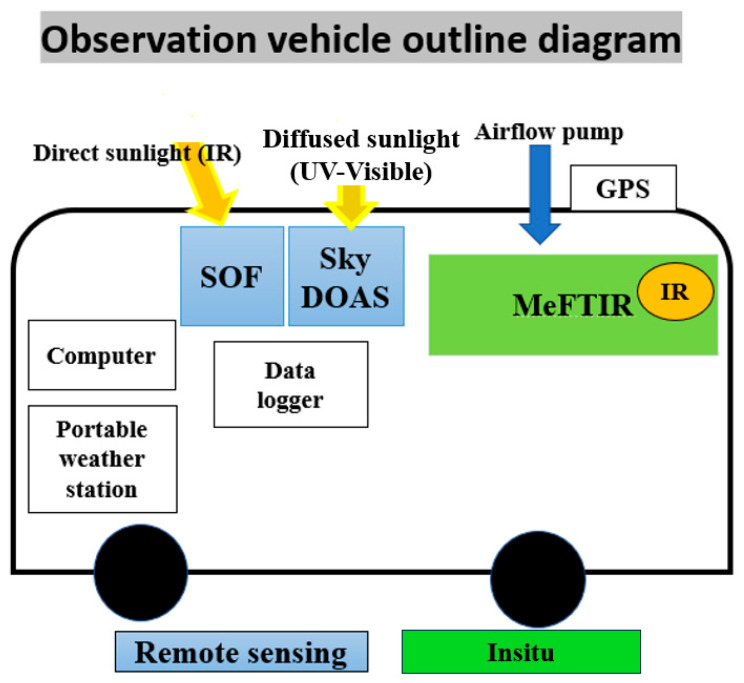
The measurement vehicle diagram.

In this study, a mobile vehicle equipped with in situ and remote sensing technology was used, including SOF, SkyDOAS, and MeFTIR as measurement techniques. The data presented in this paper were acquired using the DOAS and SOF methods, both leveraging similar principles to capture the total fluxes of industrial emission plumes rather than mere concentrations. These techniques employ open-path absorption spectroscopy to measure column concentrations, with the primary difference lying in their spectroscopic approaches. Mobile DOAS relies on ultraviolet (UV) measurements of scattered sunlight [13], whereas SOF is based on infrared (IR) measurements of direct sunlight [14]. The technology utilized in this study provides real-time alerts to communities located downwind, enhances existing emission inventory estimates, accurately measures facility-wide emissions, improves the effectiveness of regulatory policies, and showcases the feasibility and effectiveness of fence line monitoring using optical remote sensing. Fence line monitoring is a method that integrates measurement data and wind information at the boundary of a specific area to estimate the amount of emitted pollutants within that area. This method is commonly used in locations such as oil refineries, where leaks can occur from pipes and tanks. Recently, the United States Environmental Protection Agency (EPA) has been expanding the use of fence line monitoring for benzene emissions (e.g., the benzene fence line monitoring program of the U.S. EPA) [15]. In fence line monitoring, more frequent observations across various times of the day would strengthen the representativeness of the data for that day. However, depending on the weather situation and sunlight altitude, the measurement period might differ from one day to another. In general, conducting a full measurement four times around the defined boundary provides a good representation of the emitted pollutants over that area and can represent the daily average values. In this study, to ensure precise measurement, at least four full measurements were conducted daily from nearly 8:00 a.m. to 3:00 p.m. (as long as the sun was trackable). Since the influence of light scattering by solid or liquid particles in the atmosphere is important, especially near industrial complexes, to minimize the impact of scattering, measurements were conducted during periods of clear atmospheric conditions whenever possible. In addition, baseline spectra were recorded in upwind regions of the industrial complexes, where pollutant concentrations were expected to be minimal. These spectra were used to correct for baseline deviations caused by scattering effects. Also, any measurements potentially influenced by heavy particulate loads (e.g., during haze or fog) were excluded from the final calculations. This was complemented by cross-referencing with meteorological data, including visibility and particulate matter levels, when available. Figure 3 illustrates the measurement vehicle diagram and other sections in depth.

#### 2.2.1. Remote Sensing Techniques

##### Solar Occultation Flux (SOF)

The SOF technique involved recording the broadband IR spectra of the sun through a Fourier transform infrared spectrometer (FTIR) connected to a solar tracker. A telescope tracked the sun, directing its light into the spectrometer regardless of its position. The solar tracker and telescope continuously guided the sunlight into the spectrometer, and IR spectra were recorded consecutively using multivariate optimization from the solar spectrum to integrate path-integrated concentrations. Using these solar spectra, it was feasible to retrieve the path-integrated concentrations (column concentrations), using multivariate optimization, in the unit mg/m^2^, of various species between the sun and the spectrometer. The measurement from a mobile platform made it possible to circumvent leaking areas, discriminating between upwind and downwind mass fluxes. To estimate the gas flux, the column measurements were combined with wind measurements. These spectra were evaluated for absorption by molecular species in the industrial emission plume.

In more detail, SOF used a Bruker IRCube FTIR spectrometer with a spectral resolution of 0.5 cm^−1^ equipped with a solar tracker, dual InSb (indium antimonide)/MCT (Mercury Cadmium Telluride) detectors, and custom software to check the column concentration of the species. The area-integrated column of the species in the intersection between the pole and the light path could be calculated using the column concentration, the solar angle calculated from the time of measurement, and the position recorded by the GPS receiver. The species flux was calculated by multiplying the orthogonal wind velocity vector component by the area integral concentration. A detailed description of the principles of the SOF technique is given by Mellqvist et al. [16]. This study focused on the SOF measurements of total alkanes. The evaluation of alkanes and alkenes is based on a broad absorption band in the region of 2700–3000 cm^−1^. Since this band corresponds to vibrational excitations of carbon–hydrogen bonds, it is called the C-H stretch band. C-H bonds exist in most VOCs; therefore, they typically have absorption features in this band. Furthermore, all alkanes have approximately the same total absorption, i.e., the area under each absorbance spectrum, for equal mass columns. These properties facilitate the estimation of the combined absorption of any mixture of alkanes with the combination of just a few of their absorbance spectra; also, this combination estimates the total mass column of the alkane mix. For species j, the flux of the full path ($Q_{N}^{j}$) can be calculated using the following equation:

(1) $$Q_{N}^{j}=\nu_{T}\cdot \int_{P}SCL_{l}^{j}\cdot \cos\left(\theta_{1}\right)\cdot \sin\left(\alpha_{l}\right)\mathrm{d}l$$

$Q_{N}^{j}$: the flux of the full path for species j.

$\nu_{T}$: the average value of the wind speed at the plume height (m/s).

$SCL_{l}^{j}$: the average column density of species j [kg/m^2^].

θ_ɭ_ = the solar zenith angle for the light path (converted to a vertical column using the cos value).

$\alpha_{l}$: the difference between the wind direction and driving direction.

${\mathrm{d}}_{l}$: the driving distance passing through the plume.

To have an accurate measurement using SOF, it was necessary to keep the driving speed at ~25 km/h with a data interval of 2 s. The measurement data provided the optical path mass in mg/m^2^, which was multiplied by the driving distance (in meters) during the measurement, so the unit was changed to mg/m. By including the wind data (in m/s) and converting mg to kg and seconds to hours, the final measurement unit was obtained in kg/h. The SOF calibration for total alkanes was performed using a set of reference spectra acquired from controlled laboratory measurements. These measurements represented mixtures of known alkane species at various concentrations to ensure accuracy in quantifying column densities from industrial plumes. The calibration process involved matching the field spectra to these laboratory-derived references through a fitting algorithm that quantified the column amount of alkanes. Also, the determination of the “alkane-free” spectrum as a baseline was achieved by collecting spectral data upwind of the emission sources, where the air was less likely to contain significant concentrations of alkanes. These upwind measurements were used as baseline spectra for subsequent flux calculations. Therefore, this paper relied on the baseline spectra recorded in non-industrial areas upwind of the emission sources with the lowest level of pollutants during periods of minimal emissions.

##### Sky Differential Optical Absorption Spectroscopy (SkyDOAS)

DOAS works in visible and UV wavelength regions while SOF works in the IR region; therefore, there are large differences in the spectrum evaluation methods and spectroscopy. But both methods measure vertical columns, which are integrated along measurement transects and multiplied by the wind to obtain the flux. The basis of flux measurements using DOAS is the same as SOF, although there is no necessity to compensate for any slant angle observations because the telescope is always pointing towards the zenith. In contrast to SOF, the DOAS technique works in cloudy skies as well, although it is preferred to carry out the measurements in a clear sky. DOAS technology has been used in atmospheric studies using scattered sunlight and artificial light sources since the 1970s [17]. The difference between the SOF and SkyDOAS techniques is that in SkyDOAS, instead of measuring direct sunlight in the IR region, the scattered light in the UV and visible regions is measured at the zenith angle using a telescope connected by fiber to a Czerny–Turner spectrometer with a Charged Coupled Device (CCD) camera. Moreover, the data interval in SkyDOAS is around 5 s. SkyDOAS used a quartz telescope that was connected to a Czerny–Turner spectrometer with a focal length of 303 mm with a thermoelectric-cooled CCD camera of 1024 × 255 pixels. In this study, SkyDOAS was used to measure HCHO, SO_2_, and NO_2_; SO_2_, NO_2_, and HCHO are detected in the wavelength range of 310 to 325 nm, 324 to 350 nm, and 322 to 350 nm, respectively.

Similarly to SOF, the SkyDOAS calibration was conducted using the laboratory-generated reference spectra of target species. While the focus of SkyDOAS in this study was not on alkanes, the methodology involved used upwind data to establish baseline absorption spectra, ensuring that measurements downwind reflected accurate plume characteristics. The mobile DOAS measurement principle is described in detail by Galle et al. [18] and Rivera et al. [19,20]. Also, the DOAS application for SO_2_ and NO_2_ measurement is explained by Johansson et al. [21], as well as for HCHO in Johansson et al. [21,22] and Johansson et al. [23].

#### 2.2.2. In Situ Technique

##### Mobile Extraction Fourier Transform Infrared Spectrometry (MeFTIR)

Mobile extraction FTIR (MeFTIR) with tracers has been commonly used to quantify alkanes, alkenes, and VOC emissions from refineries and petrochemical industries in Europe and the United States [24]. CH_4_ and other greenhouse gasses are also detectable using this technique; hence, in this study the CH_4_ emission flux was measured using MeFTIR. The MeFTIR technique provides the flux measurement and concentration mapping of gasses independently; however, it can be used to measure VOC species and to calculate column heights in detail when it is used with SOF [23]. Transmitted light was detected by an InSb detector in the 2.5 to 5.5 µm (1800 to 4000 cm^−1^) region and an MCT detector in the 8.3 to 14.3 µm (700 to 1200 cm^−1^) region. Using the MeFTIR system, ethylene, propylene, total alkanes, CH_4_, carbon monoxide (CO), carbon dioxide (CO_2_), and NO_2_ were detectable.

All the techniques used in this study (SOF, SkyDOAS, and MeFTIR) have unique specifications for measurements; Table 1 summarizes the equipment’s specifications, such as the light source, analysis method, sensitivity, response time, etc. In addition, the requirements that should be considered during the measurements are defined in Table 2.

Ensuring accurate wind data is essential for precise flux calculations. Anemometers should be positioned at a height of 10 m, with wind speeds ranging between 1.5 and 12 m/s. Table 3 and Table 4 present the meteorological parameters, measurement times and durations, and number of observations (N) in zones S_1_ and S_2_ during the measurements, respectively.

### 2.3. Wind Data Assessment 

Wind data significantly impact flux calculations, underscoring the importance of reliable measurements. Hence, two wind anemometers were deployed during the observation periods. The first device was the YOUNG Model 05103 anemometer, complemented by the Chanju Tech WH-2300S Multi-item Meteorological Instrument for measuring the wind direction and speed. Although the vertical wind speed was not directly measured, the primary aim of this study was to calculate fluxes for the vertical plane generated by horizontally moving wind. This was achieved by scanning the plume perpendicular to its movement while the observation vehicle moved horizontally, enabling flux estimation based on horizontal wind variations.

**Figure 4:**
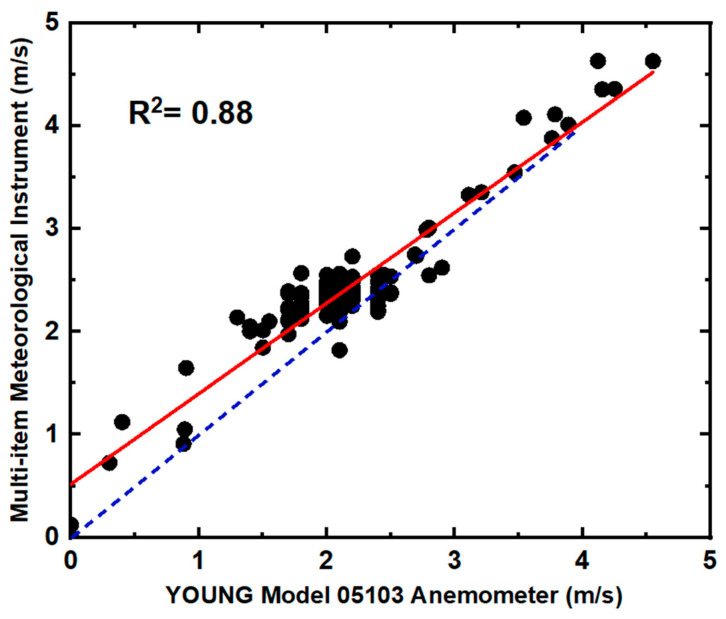
Comparison of the measured wind speed by the two anemometers (black dots), the reference relationship between the wind speeds measured by the two anemometers (red line), and the confidence intervals (blue dotted line).

To assess the performance of the two anemometers, a two-hour comparison test was conducted with both devices placed in the same location, and data were collected for analysis. Figure 4 illustrates the coefficient of determination (R^2^) between the Multi-item Meteorological Instrument and the YOUNG Model 05103 Anemometer throughout the comparison test. The R^2^ value of 0.88 indicates a strong correlation between the measured wind speeds by the two anemometers. Additionally, the results revealed that the wind speed recorded by the Multi-item Meteorological Instrument was approximately 10% higher than that of the other anemometer. As a result, only the wind speed measured by the Multi-item Meteorological Instrument was adjusted and utilized in this study following the identified discrepancy during the comparison test. The wind speed observation range, wind direction specification, and analysis interval for the Multi-item Meteorological Instrument were approximately 0–50 m/s, 0–360°, and 1 min, respectively. To ensure precise wind direction and speed data, this anemometer was positioned at heights matching those of the observation vehicle near the observation site.

### 2.4. Data Analysis

In SkyDOAS and SOF, the flux is calculated using the gradient column concentration of the measured species directly. However, in MeFTIR, instead of the column concentration, the surface concentration is calculated; therefore, the flux is calculated indirectly. The indirect flux calculation method is to multiply the alkane flux measured using SOF by the alkane value obtained using MeFTIR. Therefore, the inferred flux is calculated using the combination of SOF and MeFTIR measurements. For species (i), the inferred mass flux ($\hat{Q}j$) is calculated from the MeFTIR surface gas ratio integrated at column (p) along path (l) using the following equation:

(2) $$\hat{Q}j=\bar{Q}j\cdot \frac{1}{k}\sum_{k}\frac{\int_{p}N_{l}^{i}\mathrm{d}l}{\int_{p}N_{l}^{j}\mathrm{d}l}$$

For instance, when the alkane flux measured using SOF was 853 kg/h, the value of alkanes measured using MeFTIR was 4.6%. Hence, the calculated alkane flux value was approximately 39 kg/h (by multiplying the alkane flux value by 0.046). The uncertainty of BTEX and CH_4_ emission calculation from indirect flux calculations is greater than that of the direct measurements of alkanes.

**Figure 5:**
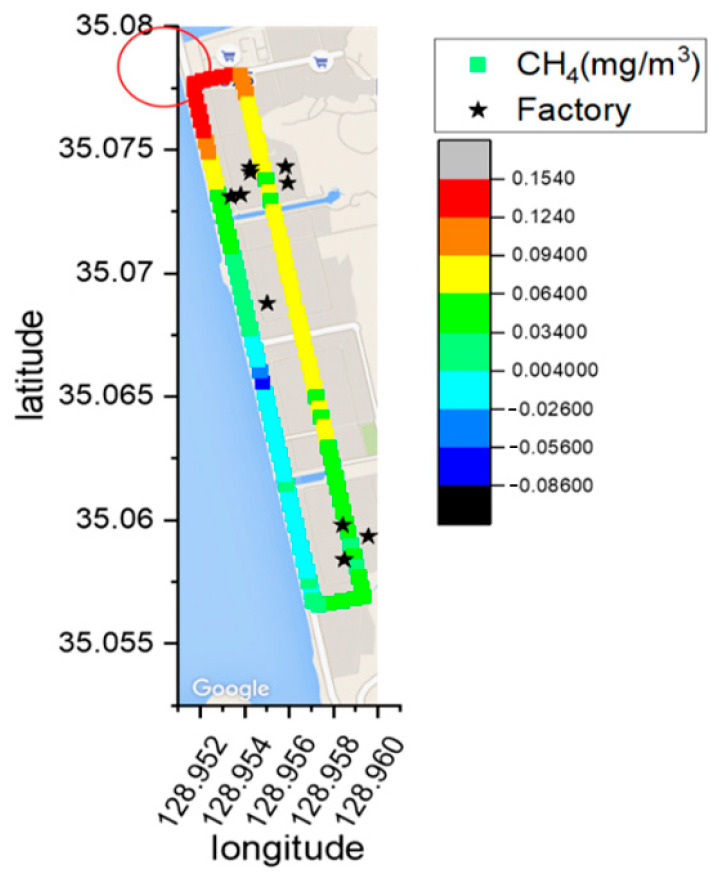
The measurement principle and the defined fence line for CH_4_ measurement in zone S_2_ (the red circle defines the expected source of CH_4_ emission).

In determining the emission source, the observed column concentrations were spatially represented on a map, considering the average wind speed and direction for each measurement. To visualize emission locations, an initial map of the measurement area was created, aiding in pinpointing areas with elevated concentrations. Utilizing the GPS inside the mobile measurement vehicle, the latitude, longitude, and concentration data were overlaid on the map. The concentration gradient was categorized into 8 levels; areas with higher concentrations were depicted in progressively redder hues. To better understand the measurement method, Figure 5 illustrates the defined fence line and measurement principles for CH_4_ measurement in zone S_2_ of the Sinpyeong Jangrim Industrial Complex within one day of measurement. The red circle at the top of Figure 5 indicates the anticipated source of CH_4_ emissions in zone S_2_, as determined by the measurement results.

### 2.5. Estimation of Pollutant Dispersion into the Surrounding Areas Using the HYSPLIT Diffusion Model

The HYSPLIT diffusion model, provided by the National Oceanic and Atmospheric Administration (NOAA) Air Resources Laboratory (ARL), is used in a variety of simulations describing the atmospheric transport, deposition, and distribution of pollutants and hazardous substances. The model calculation methodology is a hybrid between the Lagrangian approach, utilizing a moving frame of reference for the advection and diffusion calculations as the trajectories or air parcels move from their generation source, and the Eulerian method that uses a fixed three-dimensional grid as a frame of reference to compute the atmospheric pollution concentration [25]. The weather data in the HYSPLIT diffusion model are based on the Archived Global Data Assimilation System 1 (GDAS1) [26]. The diffusion model can represent forward trajectories based on the observation time at the estimated emission source. The model requires data related to the studied location (the longitude and latitude), the date of measurement (the year, month, day, and hour), and the level height.

In this study, the emission height and time were assumed to be between 10 and 50 m and 1 h, respectively. This allowed us to estimate the pollutants’ diffusion path and concentration after 1 h. An et al. [27] showed that the direction of a particle’s movements according to the wind characteristics is well identified using the HYSPLIT diffusion model.

### 2.6. Findings Comparison with PRTR System Results

The PRTR system provides information on the annual environmental releases (water, soil, and air pollution) and transfers of chemical substances industries [11]. This system focuses on emissions from large industries, with emission rates reported in kilograms per year (kg/yr). The PRTR chemical emission survey system, managed by South Korea’s Ministry of Environment’s Chemical Safety Agency, annually reports the amount of pollutant release during manufacturing processes using optical remote sensing technologies. The PRTR survey system is conducted only for large industries whose chemical consumption for manufacturing is equal to or greater than one ton per year. Emission calculations are conducted according to guidelines from South Korea’s National Institute of Environmental Sciences (NIES) guidelines, considering pollutant generation rates per hour, daily operation hours (set at 8 h), and annual operating days (5 days per week for 52 weeks). Equation (3) is utilized to estimate the annual amount of generated pollutants based on the PRTR survey system.

(3) $$Generated\;pollution=\frac{Pollutant\;amount}{h}\times \frac{40\;h}{week}\times \frac{52\;week}{1\;year}$$

## 3. Results

### 3.1. Wind Data

**Figure 6:**
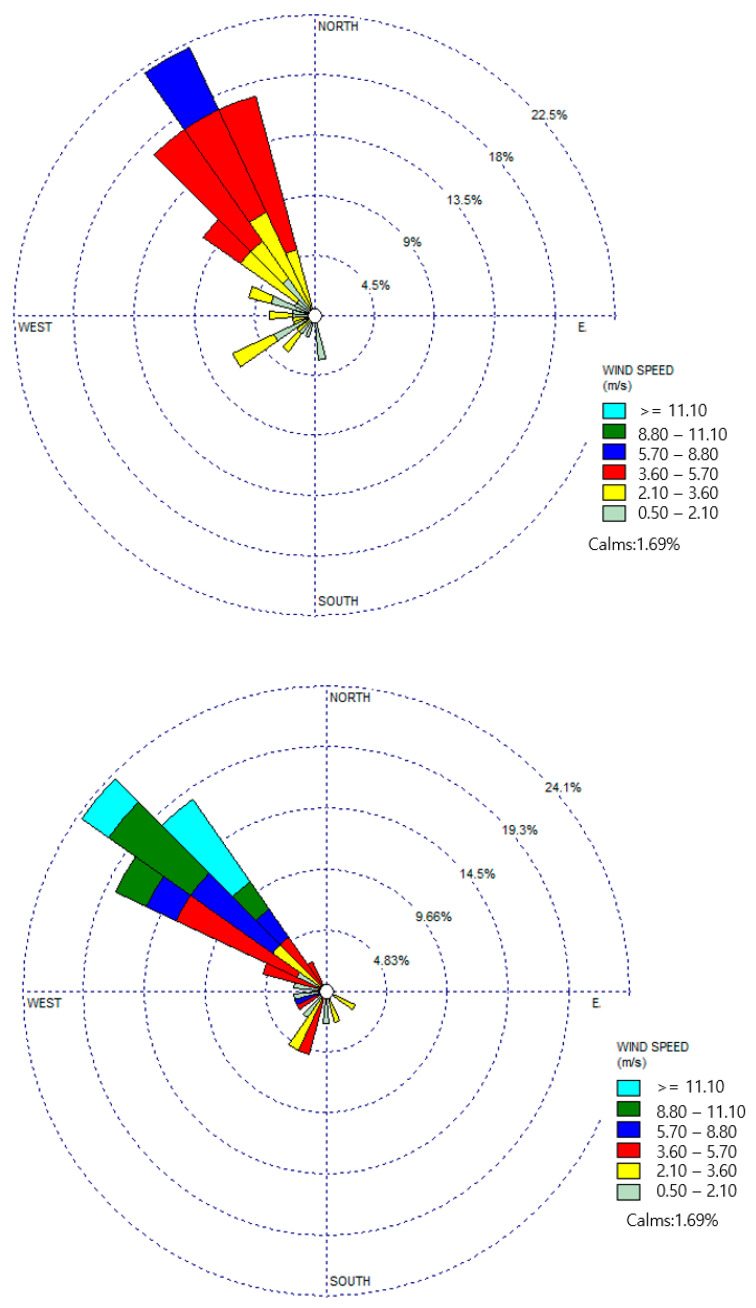
The main wind direction for zones S_1_ (on the **top**) and S_2_ (on the **bottom**) (northwesterly for both zones), and the wind speed for zones S_1_ (~2.1 m/s) and S_2_ (~3.6 m/s) during the measurement period.

Figure 6 shows the primary wind directions observed during the measurement days in zones S_1_ and S_2_. The dominant wind direction, as depicted in Figure 6, was predominantly northwesterly for both anemometers. Furthermore, the wind direction exhibited consistent alignment within a 5% margin for both devices. In zone S_1_, the wind speed generally exceeded 2.1 m/s, while in zone S_2_, it typically surpassed 3.6 m/s throughout the measurement period. Overall, the average wind speed recorded in zone S_2_ exceeded that of zone S_1_, indicating varying wind characteristics between the two zones.

The daily average wind speed and direction measured in zones S_1_ and S_2_ are presented in Table 5. It can be said that for zone S_1_ the dominant wind direction was almost uniform during the whole period of measurement (northwest); however, it was affected by the southwest and east–west wind directions on 5 and 8 November and 16 November, respectively. Moreover, the daily wind direction change was approximately 1.7 ± 0.2 m/s, which was rather small. The highest average wind speed was measured on November 9th (4.1 ± 0.3 m/s), while the lowest was measured on 16 November (1.7 ± 0.2 m/s). The highest and lowest wind direction ranges were measured on 9 November (320–337 degrees) and 16 November (98–262 degrees), respectively. In the case of zone S_2_, similarly to zone S_1_, the dominant wind direction was northwesterly in the main, except for on 5 November and 13 November with the wind directions of southwest and north–south, respectively. The minimum average wind speed was measured on 5 November (1.5 ± 0.5 m/s), whereas the maximum was measured on 9 November (5.8 ± 0.6 m/s). The greatest and lowest wind direction ranges were measured on 13 November (325–335 degrees) and 5 November (227–337 degrees), respectively.

### 3.2. The Daily Average Emission Flux Measurement for Zones S_1_ and S_2_

**Figure 7:**
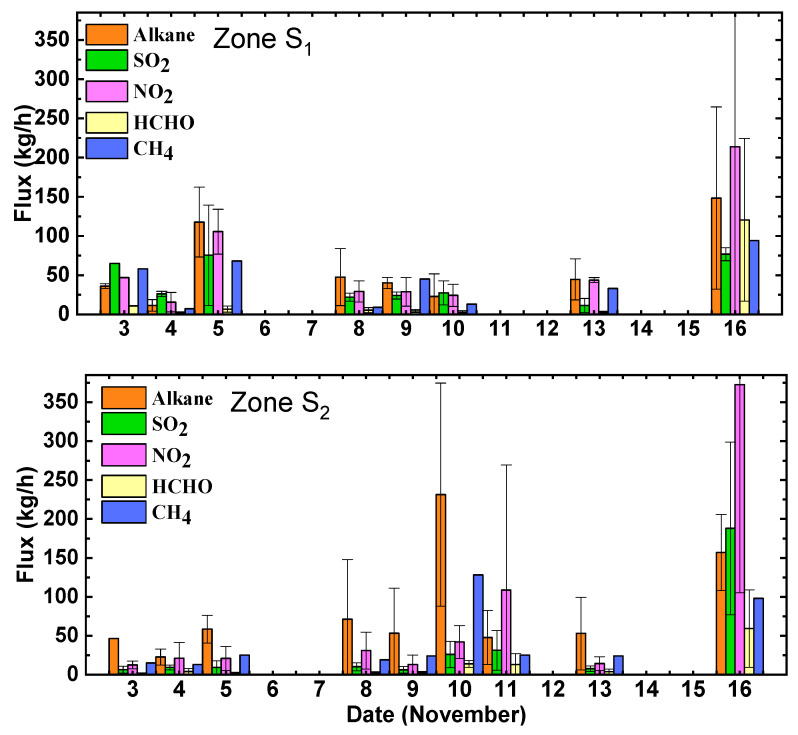
Daily average emission flux of the measured gasses for zones S_1_ (on the top) and S_2_ (on the bottom) (unit: kg/h).

By dividing the observation area (Sinpyeong Jangrim Industrial Complex) into two zones (S_1_ and S_2_), the measurements were conducted from 3 to 16 November 2020, including for 8 days in zone S_1_ and 9 days in zone S_2_ (considering the weather conditions for an effective measurement). Figure 6 summarizes the measurement results and flux report for both zones obtained using SOF, SkyDOAS, and MeFTIR during the measurement period. In the case of zone S_1_, since there were several textile and metal manufacturing industries in zone S_1_, the total daily average emission fluxes of total alkanes, SO_2_, NO_2_, HCHO, and CH_4_ were 69.9 ± 71.6 kg/h, 36.4 ± 31.6 kg/h, 64.8 ± 100.8 kg/h, 21.2 ± 54.0 kg/h, and 41.0 ± 33.0 kg/h, respectively. The highest average emission of whole gasses was measured on 16 and 5 November in zone S_1_, with the maximum values of NO_2_ (more than 200 kg/h) and total alkanes (roughly 150 kg/h) measured on 16 November. In contrast, the minimum values were measured on 4 November, with the lowest values of HCHO (3 kg/h) and total alkanes (almost 12 kg/h). Except for 16 November, during the entire measurement period, HCHO showed the lowest values among the other measured gasses. In zone S_2_, which mainly included mechanical equipment and metal manufacturing, the total daily average emission flux for total alkanes, SO_2_, NO_2_, HCHO, and CH_4_ was 84.1 ± 85.8 kg/h, 31.4 ± 63.4 kg/h, 66.3 ± 138.2 kg/h, 10 ± 22.4 kg/h, and 41.0 ± 41.6 kg/h, respectively, indicating a higher total alkane emission flux compared to zone S_1_. In the case of SO_2_ and NO_2_, the emission flux in zone S_2_ was almost similar to zone S_1_, whereas HCHO showed higher values in zone S_1_ (almost doubled). According to Figure 7, in zone S_2_ the highest emissions were measured on 16 and 10 November, with the maximum levels of NO_2_ (375 kg/h) and total alkanes (235 kg/h), respectively, while the minimum emissions were measured on 4 and 3 November, with the lowest values of HCHO (less than 1 kg/h) and SO_2_ (almost 1 kg/h). During whole days of measurements, HCHO showed the lowest emission flux compared to the other pollutants.

### 3.3. The Average Emission Flux During the Entire Period of Measurement in Zones S_1_ and S_2_

**Figure 8:**
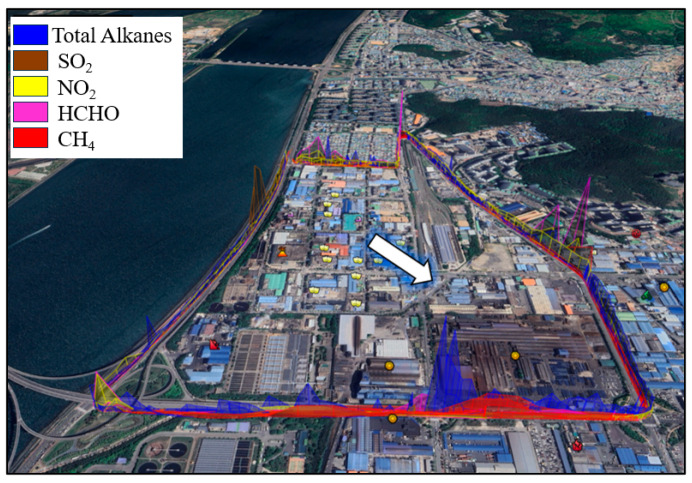
The average emission flux during the entire period of measurement in zone S_1_ (the white arrow indicates the dominant wind direction during the observation).

Figure 8 shows the average emission flux of total alkanes, SO_2_, NO_2_, CH_4_, and HCHO based on the measured gasses over the 8-day observation period in zone S_1_. The dominant wind direction, indicated by white arrows on each map, consistently points northwesterly. Given the residential areas situated to the north, northeast, and southeast of zone S_1_, it is expected that emissions from pollution sources will impact downwind residential areas. In zone S_1_, textile and apparel industries were identified as primary sources of CH_4_, NH_3_, and toluene in this zone. Moreover, metal manufacturing industries are significant emitters of HCHO, CH_4_, NH_3_, sulfuric acid (H_2_SO_4_), benzene, toluene, xylene, dichloromethane, and n-hexane. Observations revealed that for SO_2_ and CH_4_, emission levels followed a similar pattern along the measurement path, except for peaks in the northwest and north for SO_2_ and the south for CH_4_. Total alkane emissions exhibited a fluctuating distribution, with lower emissions in the west and northeast and higher fluxes in the south, north, and southeast, peaking in the south. NO_2_ emissions were minimal in the west and southeast of zone S_1_, while lower HCHO emission fluxes were recorded in the west, northeast, and southeast, contrasting with higher levels in the east, north, and south regions.

Generally, elevated levels of the measured gasses were observed in the south, southeast, and east, with peaks noted in total alkanes (in the south) and HCHO (in the east). Unlike CH_4_, NO_2_ showed a lower emission flux in the south; however, higher values were measured in the north, east, and northeast. Except for SO_2_, which peaked in the west, minimum values of other pollutants were found on this side of zone S_1_, which is adjacent to the coast. On the contrary, higher values of the measured pollutants, specifically total alkanes and CH_4_, were found in the downwind area of zone S_1_.

**Figure 9:**
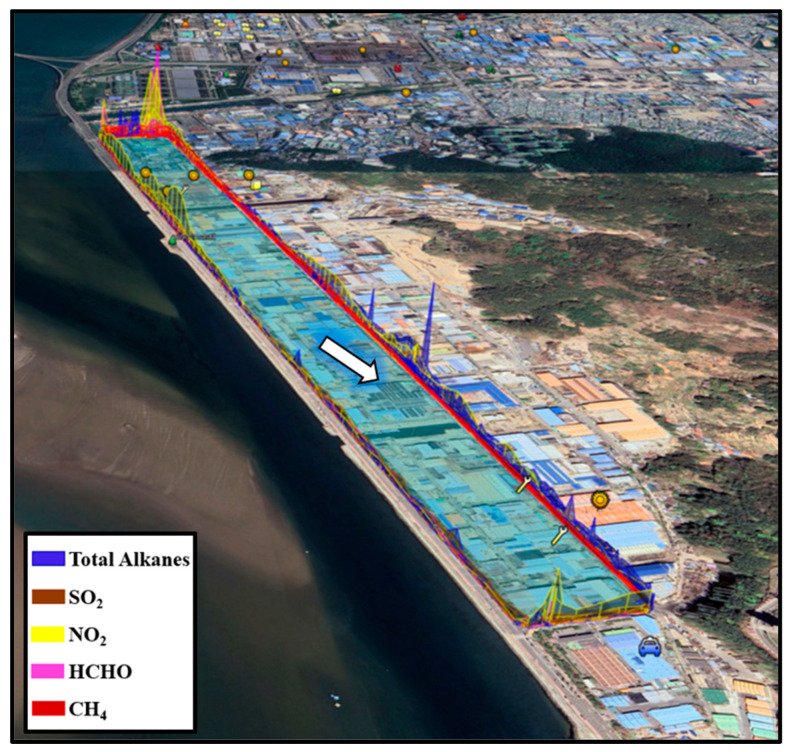
The results of whole days of measurement based on the substances in zone S_2_ (the white arrow indicates the dominant wind direction during the observation).

Figure 9 represents the average emission flux of total alkanes, SO_2_, NO_2_, HCHO, and CH_4_ measurements over the 9-day observation in zone S_2_. Once more, the prevailing wind direction in this zone is illustrated by white arrows. While HCHO and CH_4_ emission fluxes remained relatively consistent across the measured areas (with occasional peaks in the north), total alkanes, SO_2_, and NO_2_ exhibited significant fluctuations along the measurement path. According to the data in Figure 9, lower total alkane values were recorded in the west, with higher values observed in the north, east, and southeast. The NO_2_ emission flux peaked in the north, northwest, east, and south regions. HCHO levels were predominantly low along the measurement path, except for two peaks in the north. Conversely, for SO_2_, minimal values were found in the northeast, east, and southeast. The highest CH_4_ values were identified in the north. Generally, slightly lower values of all measured gasses were found in the west, which is adjacent to the sea. Maximum pollution levels were identified downwind of zone S_2_, which is southeast, east, and south; however, NO_2_, HCHO, and CH_4_ peaked in the upwind area (northwest and north). According to the map, zone S_2_ is surrounded by a hill to the east, which might influence the stagnation of air pollutants in this zone.

### 3.4. Pollutant Dispersion Estimation for Zones S_1_ and S_2_ Using the HYSPLIT Diffusion Model

**Figure 10:**
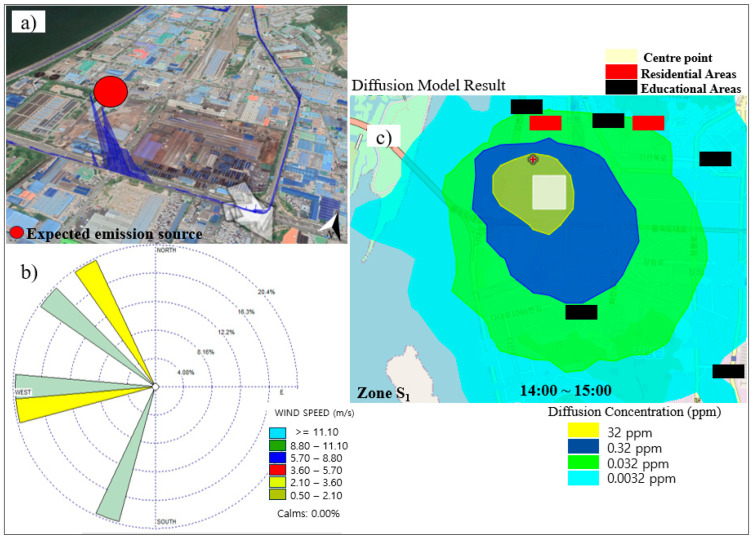
Total alkane dispersion estimation based on the HYSPLIT diffusion model in zone S_1_ (5 November 2020, at 1:57 p.m., 2:17 p.m., and 2:42 p.m.). (**a**) Expected emission source according to the observed peak, (**b**) wind speed and direction data, and (**c**) the HYSPLIT diffusion model results.

To approximate the distribution of emitted pollutants into the surrounding areas, the HYSPLIT diffusion model was applied on 5 November (for total alkanes), 2020, for almost an hour (at 1:57 p.m., 2:17 p.m., and 2:42 p.m. for total alkanes) in zone S_1_. Figure 10 represents the expected emission source according to the observed peak, wind speed, wind direction, and total alkane dispersion estimation based on the HYSPLIT diffusion model in zone S_1_. As shown in Figure 10, the total alkane emission source in zone S_1_ on 5 November was expected to be located in the south, depicting a peak in this region of the measurement area. The dominant wind direction was almost northwesterly, and the wind speed did not exceed ~3.0 m/s. The results of the diffusion model confirmed the influence of measured pollutants (total alkanes) on the adjacent residential areas with values less than 0.032 ppm. The results showed that when the measured average emission flux of total alkanes at the time of observation was 32.0 ± 20.5 kg/h in zone S_1_, in the adjacent residential areas, the average estimated emission was about 0.003 to 0.03 ppm based on the HYSPLIT diffusion model estimation.

**Figure 11:**
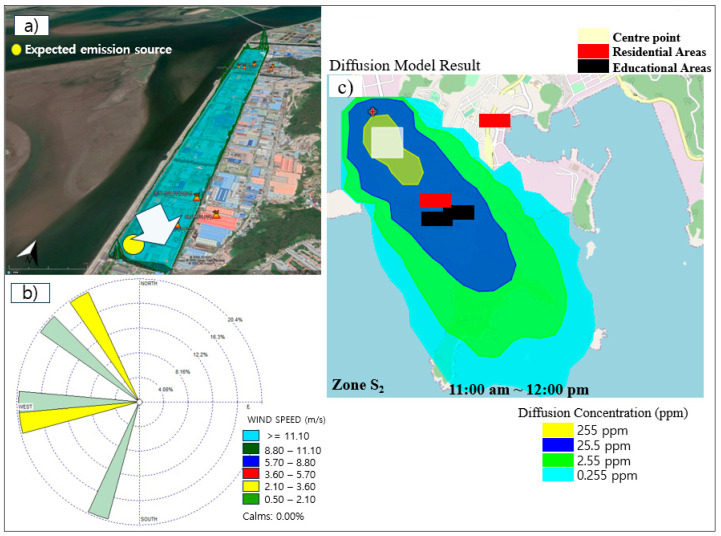
NO_2_ dispersion estimation based on the HYSPLIT diffusion model in zone S_2_ (16 November, at 10:41 a.m. and 12:00 p.m.). (**a**) Expected emission source according to the observed peak, (**b**) wind speed and direction data, and (**c**) the HYSPLIT diffusion model results.

Figure 11 represents the expected emission source according to the observed peak, wind speed, wind direction, and NO_2_ dispersion estimation based on the HYSPLIT diffusion model in zone S_2_ (16 November 2020, at 10:41 a.m. and 12:00 p.m.). As is shown, a peak was observed on the south side of zone S_2_, demonstrating the location of the expected NO_2_ emission source. In addition, the wind speed was estimated to be less than 3.0 m/s, with the dominant direction of northwest and west. The HYSPLIT diffusion model result confirmed that the downwind residential areas were influenced by NO_2_ emission pollution with values less than 25.5 ppm. For NO_2_, when the measured average emission flux was 254.6 ± 65.0 kg/h at the time of observation, in the adjacent residential areas, the average estimated emission was approximately 0.25 to 25.5 ppm according to the HYSPLIT diffusion model estimation.

## 4. Results Comparison with the PRTR Survey System

The PRTR survey system reports items such as the annual air pollution emissions including total alkanes, NO_2_, SO_2_, and HCHO from large industries. In 2020, there were a total of 497 operating divisions in Busan’s Sinpyeong Jangrim Industrial Complex. Among these, 29 large industries reported their industrial air pollution emissions to the government through the PRTR survey.

Based on the measurements from this study, the total daily average emission fluxes of total alkanes, NO_2_, SO_2_, and HCHO in zone S_1_ were 69.9 ± 71.6 kg/h, 64.8 ± 100.8 kg/h, 36.4 ± 31.6 kg/h, and 21.2 ± 54.0 kg/h, respectively. Using Equation (3), the sum of the emission fluxes of the measured gasses was calculated as 192.3 kg/h. When converted to an 8 h day, 5 days a week, and 52 weeks a year, the total emission flux was calculated as 399,984 kg/yr for zone S_1_. The total daily average emission fluxes of total alkanes, NO_2_, SO_2_, and HCHO in zone S_2_ were 84.1 ± 85.8 kg/h, 66.3 ± 138.2 kg/h, 31.4 ± 63.4 kg/h, and 10.0 ± 22.4 kg/h, respectively. Based on Equation 3, the sum of the emission fluxes of the measured gasses was 191.8 kg/h, with an annual flux emission of 398,944 kg/yr. Table 6 compares the PRTR survey results for the Sinpyeong Jangrim Industrial Complex reported by Korea’s government, which were 16,443 kg/yr for zone S_1_ and 80,956 kg/yr for zone S_2_. As is shown, the total flux emissions measured in this study were roughly 24.3 and 4.9 times higher than the PRTR-reported results in zones S_1_ and S_2_, respectively.

## 5. Discussion

### 5.1. Zone S_1_

In the Sinpyeong Jangrim Industrial Complex, where residential and industrial zones coexist, the inadequate monitoring of pollutant emissions can pose significant health risks to residents. Therefore, fence line monitoring and establishing a regular assessment system for emitted pollutants is imperative in such areas. The mobile optical remote sensing equipment employed in this study allows for continuous monitoring, operating without spatial limitations as long as there is a path to sunlight. By creating a defined fence line around the study area and utilizing this equipment to monitor emissions in real time from each source, the main emission sources can be identified by assessing the characteristics of industries within the designated perimeter, including the location, manufacturing processes, emission sources, wind speed, and wind direction.

Given that the wind direction and speed play a crucial role in air pollution dispersion, they must be key factors when evaluating areas affected by pollutants. In zone S_1_, the prevalent northwesterly wind was influenced by a southwest wind on 5 and 8 November, and an east–west wind on November 16th, with a daily wind direction change of 1.7 ± 0.2 m/s, a relatively modest variation.

The residential areas nearest to the S_1_ industrial zone include R_1_ (north and northeast), R_2_ (east), and R_3_ (south and southeast). With the predominant northwesterly wind direction observed by the anemometer, the R_3_ residential area, located downwind, is expected to be notably impacted by pollutants emitted from S_1_ compared to other residential zones [24]. Additionally, based on the wind direction, the R_2_ residential area may also be affected by emissions from the S_1_ industrial zone. R_4_ and R_5_, situated downwind of S_1_, might experience pollutant exposure only during periods of high wind speeds due to the greater distance separating these residential areas from S_1_.

In zone S_1_, the average wind speed and the wind speed range measured by the installed anemometer were 2.7 ± 0.3 m/s and 1.5 to 4.4 m/s during the entire period of measurement, respectively. According to the measurement equipment principles, the minimum wind speed for accurate optical remote sensing observation must be 1.5 m/s or more, and the observation accuracy might be reduced with a stable atmospheric condition or low wind speed. The lowest wind speed was measured on 16 November (1.7 ± 0.2 m/s), which was accompanied by the highest levels of pollutants due to the atmospheric stagnation and low wind speed. The lowest levels of pollutants were found on 4, 8, 9, and 10 November, with the highest wind speed on 9 and 4 November (4.1 ± 0.3 m/s and 3.7 ± 0.3 m/s), respectively, which can emphasize the role of the wind speed in the reduction in air pollutants. During whole days of measurement, the average flux of total alkanes, SO_2_, NO_2_, HCHO, and CH_4_ was 69.9 ± 91.6 kg/h, 36.4 ± 31.6 kg/h, 64.8 ± 100.8 kg/h, 21.2 ± 54 kg/h, and 41.0 ± 33.0 kg/h, respectively. However, regarding the average daily flux variations, all the measured pollutants showed a high emission flux on Nov 16th.

On 16 November, the average flux of total alkanes was 148.3 ± 116.2 kg/h, nearly twice the average value (69.9 ± 91.6 kg/h). The average fluxes for SO_2_ and NO_2_ on 16 November were 76.8 ± 8.3 kg/h and 213.8 ± 222.6 kg/h, which were about two and more than three times higher than the average values of 36.4 ± 31.6 and 64.8 ± 100.8 kg/h, respectively. In the case of HCHO and CH_4_, the measured average fluxes on 16 November were 120.5 ± 103.7 kg/h and 94 kg/h, which were about six times and two times higher than the average total values of 21.2 ± 54 kg/h and 41.0 ± 33.0 kg/h, respectively. The considerable difference in the average flux emission on November 16th compared to the other days is mainly due to the significantly low wind speed (1.7 ± 0.2 m/s) and the accumulation of pollutants in response to atmospheric stagnation. Additionally, on 16 November 2020, being a Monday and the first working day of the week, emissions from automobiles and traffic could have contributed to atmospheric stagnation, potentially leading to a decline in air quality. Despite the low wind speed and elevated emission flux of the measured gasses, the prevailing east–west wind direction suggests that the impact of air pollutants on residential areas may be minimal. Therefore, a low wind speed, atmospheric stagnation, emissions from vehicles, and traffic-related effects can be considered the primary factors influencing the high average flux of measured gasses on 16 November.

To pinpoint the primary cause of the heightened pollutant concentration and draw a more precise conclusion, it is recommended to extend the number of observation days in this area. The R_3_ residential area is anticipated to have experienced the most significant impact from the measured gasses on November 9th, November 4th, and November 3rd due to the prevailing northwesterly wind direction and higher wind speeds (4.1 ± 0.3 m/s, 3.7 ± 0.3 m/s, and 3.0 ± 0.4 m/s, respectively). Conversely, the most adverse effects of the measured air pollutants on the R_2_ and R_1_ residential areas are forecasted to have occurred on November 8th, considering the southwesterly wind direction.

The Sinpyeong Jangrim Industrial Complex encompasses both large-scale operations (106 and 74 operating divisions for metal manufacturing and mechanical equipment) and various small-scale industries such as food and beverage production, textile and apparel production, electrical equipment, transportation equipment, automobile manufacturing, rubber and plastic production (64, 46, 40, 29, 27, and 25 operating divisions, respectively), and others. Given that metal manufacturing (21%) and mechanical equipment (15%) are the dominant industries in the complex, they are expected to significantly impact the surrounding air quality. On the other hand, the majority of industries within the complex are small businesses that often do not receive adequate monitoring due to their impact on air quality being underestimated or overlooked. Even though emissions from small-scale industries are typically low and their emission control equipment is insufficient, the emissions from these businesses can significantly alter local air quality. Given that the focus is usually on large-scale industries, emissions from small businesses are not closely monitored.

Alkane compounds play a significant role in chemical industries, particularly in crude oil refining and energy generation, where single alkane compounds like CH_4_ or mixtures of alkane compounds such as gasoline and diesel are utilized based on specific purposes [28]. CH_4_ is generated through the breakdown of organic matter in the absence of oxygen. Global CH_4_ emissions stem from both natural (40%) and anthropogenic sources (60%), with anthropogenic sources including agriculture, energy production, industrial activities, and waste management [29]. In zone S_1_, additional sources of CH_4_ include textile industries, clothing factories, and traffic.

In this study, the equipment was used to estimate fluxes based on the concept of fence line monitoring. This involved detecting concentration changes in the plumes emitted from within the fence line boundary as they moved outward and calculating fluxes by integrating this information with meteorological data. One of the key strengths—and limitations—of this method is its ability to exclude background concentrations unrelated to emissions within the fence line. For CH_4_, with particles with a long lifetime that were not currently being emitted, the concentration gradients inside and outside the fence line were effectively equal, meaning CH_4_ did not contribute to the calculated flux. Consequently, only the methane actively being emitted was captured in the flux estimation.

SO_2_ and NO_2_, recognized as primary conventional air pollutants, are predominantly emitted by iron and steel factories within the Sinpyeong Jangrim Industrial Complex [30]. HCHO in the complex primarily originates from metal manufacturing processes, with other sources including resin used in composite wood product manufacturing, household goods production (such as adhesives, paints, and coatings), preservatives in certain pharmaceutical manufacturing, and paper product manufacturing [31]. Within zone S_1_, major sources of NH_3_ and toluene were textile and clothing industries. Apart from agricultural activities, non-agricultural processes like urban waste management (wastewater treatment and solid waste disposal), fossil fuel combustion, vehicle emissions, and even urban green spaces can release substantial amounts of NH_3_ into the atmosphere [32]. H_2_SO_4_, commonly employed in chemical industries, fertilizer production, mineral processing, oil refining, wastewater treatment, and chemical synthesis, was predominantly emitted from chemical and pharmaceutical manufacturing divisions in zone S_1_. Given that metal manufacturing and mechanical equipment industries are prominent in zone S_1_ of the Sinpyeong Jangrim Industrial Complex, the elevated levels of total alkanes, NO_2_, and SO_2_ in the area appear to be logically associated with these industrial activities.

### 5.2. Zone S_2_

Similarly to zone S_1_, a comprehensive analysis of the dominant wind direction and speed, industry characteristics within the defined fence line, and emission types, along with comparing the results with the PRTR survey system, aided in identifying the primary emission sources in zone S_2_. Compared to zone S_1_, more industrial facilities are located in zone S_2_ and the major industries in this zone are metal and mechanical manufacturing industries; therefore, HCHO, CH_4_, dichloromethane, n-hexane, NH_3_, H_2_SO_4_, benzene, toluene, and xylene were expected to highly affect the air quality in this zone. The wind direction and speed play vital roles in pollutant dispersion and air pollution mitigation. Elminir [33] observed a significant negative correlation between the total urban air pollution concentration and the wind speed; in addition, Yadav et al. [34] noted a reduction in CO and NO_X_ levels with an increasing wind speed. Throughout the nine-day measurement period in zone S_2_, the prevailing wind direction was predominantly northwesterly, except on 5 and 13 November, when the wind direction shifted to southwest and northwest, respectively. Consequently, based on the understanding of pollutant dispersion by wind (which follows the wind speed and direction), it is reasonable to expect higher pollutant levels in the south, east, and southeast areas compared to other sections of the S_2_ zone. Nevertheless, other factors such as the atmosphere temperature and humidity, boundary layer, topography, industrial distribution, and additional environmental influences can also affect pollutant levels.

In zone S_2_, the average wind speed over the measurement period was 3.7 ± 0.5 m/s, with a range of 1.5 to 8.4 m/s, indicating slightly higher speeds than those in S_1_. Days with lower wind speeds, such as 5, 10, and 16 November, at 1.5 ± 0.5 m/s, 3.2 ± 0.6 m/s, and 3.2 ± 0.6 m/s, respectively, may have experienced atmospheric stagnation, leading to elevated pollution levels compared to other days. Notably, the maximum daily average flux of measured gasses occurred on 16 November with a peak in NO_2_ (approximately 375 kg/h) and 10 November with the highest total alkane value (230 kg/h) due to reduced wind speeds. Despite the minimum wind speed on 5 November, a lower daily average flux was found on this day compared to 10 and 16 November. The wind speed on 5 November approximately equaled the lowest wind speed required for an accurate measurement (1.5 m/s or more) and decreased the influence of air-borne gasses on the R_3_ residential area, which is already affected by pollutants generated in S_1_. Moreover, the dominant wind direction on 5 November was southwest, which indicates a lower influence on the R_5_ residential area located southeast of the S_2_ industrial area. Though the R_4_ residential area is located downwind of the S_2_ industrial zone, due to the long distance between S_2_ and R_4_ and the presence of a low-height mountain on the east side of S_2_, R_4_ is less influenced by the pollutants from S_2_. However, the residential areas located on the mentioned mountain are directly affected by the S_2_-generated pollutants.

In zone S_2_, the total average flux during the entire period of measurement for total alkanes, SO_2_, NO_2_, HCHO, and CH_4_ was 84.1 ± 85.8 kg/h, 31.4 ± 63.4 kg/h, 66.3 ± 138.2 kg/h, 10 ± 22.4 kg/h, and 41.0 ± 41.6 kg/h, respectively. The total average flux of total alkanes in zone S_2_ was higher than in S_1_, whereas the total average flux of HCHO in zone S_1_ was nearly twice the one in S_2_. SO_2_, NO_2_, and CH_4_ showed roughly similar values in both industrial zones. Zone S_2_ mainly consists of metal and mechanical industries; hence, it was estimated we would observe higher values of total alkenes, benzene, toluene, xylene, CH_4_, dichloromethane, n-hexane, NH_3_, and H_2_SO_4_ in this zone, in comparison to S_1_. Further, the number of factories emitting total alkanes in zone S_2_ was higher than in S_1_, which can explain the difference in the total average flux of total alkanes between the two zones. Similarly to zone S_1_, the average daily flux of all measured gasses exhibited heightened values on 16 November in zone S_2_. On this day, the total daily average flux for total alkanes, SO_2_, NO_2_, and HCHO was notably elevated at 157.0 ± 8.7 kg/h, 187.9 ± 110.9 kg/h, 372.4 ± 267.0 kg/h, and 59.2 ± 49.8 kg/h, respectively. These values represent roughly twice the total average emission for total alkanes and approximately six times the total average emission for SO_2_, NO_2_, and HCHO compared to the entire measurement period. The relatively low average wind speed on this day (3.2 ± 0.6 m/s) underscores the potential role of a reduced wind speed in atmospheric stagnation and pollutant buildup. Notably, as November 16th, 2020, was Monday, the busiest day of the week with heightened traffic congestion, increased emissions from traffic could also have contributed to the elevated pollutant levels in zone S_2_.

Given that the primary industries in zone S_2_ are metal and mechanical manufacturing, SO_2_ and NO_2_ were assumed to be the primary pollutants, emitted from iron and steel factories in this area of the Sinpyeong Jangrim Industrial Complex [35]. Metal manufacturing, with a greater number of divisions compared to S_1_, was identified as the main source of HCHO in S_2_, with minor contributions from the pharmaceutical industry, paper product manufacturing, and paint factories [36]. While NH_3_ and toluene predominantly emanate from clothing and fabric factories, in zone S_2_, vehicular emissions, fossil fuel combustion, and urban waste may also act as additional sources of NH_3_ [37]. In general, akin to zone S_1_, the presence of metal manufacturing and mechanical equipment in zone S_2_ of the Sinpyeong Jangrim Industrial Complex made the elevated levels of total alkanes, NO_2_, and SO_2_ quite foreseeable.

### 5.3. Pollutant Dispersion Estimation Using the HYSPLIT Diffusion Model

The HYSPLIT diffusion model was utilized in this study to estimate the dispersal of pollutants into the atmosphere. Observations were conducted based on the following specific measurement criteria: an emission flux ranging from 1 kg/h upwards at 10 to 50 m heights, and an emission duration of 1 h. This facilitated the prediction of diffusion patterns and pollutant concentrations after one hour. The model indicated the forward trajectory from the emission source point in both zones based on the observation time. For zone S_1_, the model estimated that total alkanes affected adjacent residential areas with concentrations below 0.032 ppm on the estimation day. Similarly, in zone S_2_, the model showed that downwind residential areas were influenced by NO_2_ emissions with concentrations below 25.5 ppm on the same day. Consistent with our results, the HYSPLIT diffusion model highlighted the predominant direction of pollutant dispersion, mainly from the northwest to the southeast, impacting downwind locations.

Since the HYSPLIT diffusion model is based on chimney emissions, the measured emission flux and the diffusion concentration estimated by the HYSPLIT diffusion model might show some differences. In addition, the HYSPLIT’s minimum time step is 1 min, so the model cannot be used for transport less than the distance it takes for the pollutant to move in 1 min. However, the meteorology may not adequately represent the transport or dispersion at the point of release regardless of the model time step. The HYSPLIT diffusion model uses its own meteorological data; nonetheless, a comparison of the measured meteorological data in this study with the ones provided by the HYSPLIT diffusion model showed a minimal difference. Therefore, this dissimilarity and its probable influence on the model’s prediction was ignored. 

### 5.4. Results of Comparison with the PRTR Survey System

The Sinpyeong Jangrim Industrial Complex comprises both small and large industries, with the majority of businesses in the complex being small and having lower annual air pollution emissions compared to the large ones. Although large businesses emit significant levels of pollution into the atmosphere (specifically metal manufacturing and mechanical equipment, which, based on the PRTR survey system, are the main sources of total alkane emission in the area), their number is limited [11]. On the other hand, while the contribution of small businesses to air pollution is lower compared to large businesses, the sheer number of small businesses can have a notable influence on atmospheric pollution. In this study, among 497 operating divisions in Busan’s Sinpyeong Jangrim Industrial Complex, 29 large industries reported their industrial air pollution emissions to the government through the PRTR survey. Therefore, it can be said that the role of small businesses in the annual air pollution emission reported by PRTRs was ignored.

A comparison of our findings with the PRTR survey results revealed that the total flux emissions measured in this study were nearly 24.3 and 4.9 times higher than the PRTR-reported results in zones S_1_ and S_2_, respectively. This highlights the importance of a large number of small industries in exacerbating air pollution in an industrial area.

## 6. Conclusions

This study employed a combination of mobile remote sensing and in situ technologies to assess the pollutant emission flux and identify emission sources within an industrial complex (the Sinpyeong Jangrim Industrial Complex) situated in Busan City, South Korea. By utilizing the fence line monitoring approach, the emission flux of pollutants was quantified in the Sinpyeong Jangrim Industrial Complex.

We identified the wind direction and speed as crucial factors influencing the dispersion of air pollutants. The mobile measurement technology employed facilitated the identification of primary emission sources by pinpointing areas with a high emission flux and analyzing wind data through repeated measurements along defined fence lines.

The Sinpyeong Jangrim Industrial Complex primarily comprises small businesses. However, these industries often receive insufficient recognition, resulting in the underestimation or neglect of their emission loads. A comparison of the PRTR survey system results with this study showed that despite the fact pollutant emissions from small-scale industries are typically low and their emission control equipment is often inadequate, the emissions from these small businesses can significantly affect local air quality. Since the focus is typically on large-scale industries, emissions from small businesses are frequently not thoroughly monitored. This lack of monitoring can lead to the dispersion of pollutants into nearby residential areas, potentially impacting air quality.

The HYSPLIT diffusion model further confirmed the detrimental effects of industrial emissions from the Sinpyeong Jangrim Industrial Complex on neighboring residential areas, underscoring the need for stricter preventive measures by governmental bodies. The continuous monitoring of gasses undergoing volatilization is thus essential. Despite the limited measurement period, the data collected in this study offer valuable insights into emission sources and the pollutant emission flux, emphasizing the importance of integrating fence line monitoring data with remote sensing, in situ observations, meteorological data, and residential concentration measurements to enhance emission monitoring and refine local emission mitigation strategies.

Future studies aim to extend the measurement period to provide more precise assessments of industrial complexes’ impact on residential areas in Busan City, enhancing the efficacy of emission reduction policies at a local level.

## Figures and Tables

**Table 1:** The equipment’s specifications for SOF, SkyDOAS, and MeFTIR measurement.

Measurement Technique	Equipment Name	Light Source	Measured Compounds	Analysis Method	Measured Unit	Inference Data	Sensitivity	Flux Limit	Response Time
Remote Sensing	SOF	Direct sunlight (IR)	Total alkanes, alkenes, NH_3_	FTIR	Vertical path-integrated column [mg/m^2^]	Mass flux [kg/h]	0.1~5 mg/m^2^	0.2~1 kg/h	1~5 s
	SkyDOAS	Scattered sunlight (UV–Vis)	NO_2_, SO_2_, HCHO	DOAS	Vertical path-integrated column [mg/m^2^]	Mass flux [kg/h]	0.1~5 mg/m^2^	1 kg/h	1~5 s
In Situ	MeFTIR	IR	Alkanes, CH_4_, C_2_H_4_, C_3_H_6_, C_4_H_8_, NH_3_, CO, CO_2_, N_2_O	FTIR	Concentration [mg/m^3^]	Mass flux [kg/h], column height information [m]	1~10 ppbv *	0.2~2 kg/h	5~15 s

* ppbv: parts per billion by volume.

**Table 2:** Considered requirements during measurements.

Title	Requirement
Data gaps in the plume	<20%
Upwind flux	<50%
Solar angle	>20°
Vehicle stops	<20%
Wind speed	1.5–12 m/s

**Table 3:** Meteorological parameters, measurement times and durations, and number of observations (N) in zone S_1_ during the measurements.

Zone S_1_	Meteorological Parameters	Measurement Time	SOF	SkyDOAS			MeFTIR
			Alkanes	SO_2_	NO_2_	HCHO	CH_4_
Date	Weather Condition	Measurement Duration	N	N	N	N	N
3 November	Ave Temp: 9.7 °C Weather Condition: Sunny Ave Humidity: 25.37%	08:30 a.m.–10:30 a.m. (2 h)	4	4	4	4	4
4 November	Ave Temp: 8.7 °C Weather Condition: Sunny Ave Humidity: 22.45%	12:30 p.m.–02:30 p.m. (2 h)	4	4	4	4	4
5 November	Ave Temp: 12.0 °C Weather Condition: Sunny Ave Humidity: 35.47%	08:30 a.m.–10:30 a.m. (2 h)	4	4	4	4	4
8 November	Ave Temp: 14.1 °C Weather Condition: Sunny Ave Humidity: 28.27%	09:00 a.m.–11:00 a.m. (2 h)	4	4	4	4	4
9 November	Ave Temp: 9.2 °C Weather Condition: Sunny Ave Humidity: 15.24%	09:00 a.m.–11:00 a.m. (2 h)	4	4	4	4	4
10 November	Ave Temp: 11.0 °C Weather Condition: Sunny Ave Humidity:26.69%	09:00 a.m.–11:30 a.m. (2.5 h)	5	5	5	5	4
13 November	Ave Temp: 15.6 °C Weather Condition: Sunny Ave Humidity: 29.36%	08:30 a.m.–10:30 a.m. (2 h)	4	4	4	4	4
16 November	Ave Temp: 16.4 °C Weather Condition: Cloudy Ave Humidity: 33.58%	09:30 a.m.–11:30 a.m. (2 h)	5	4	4	4	4
Total	-	16.5	34	33	33	33	32

**Table 4:** Meteorological parameters, measurement times and durations, and number of observations (N) in zone S_2_ during the measurements.

Zone S_2_	Meteorological Parameters	Measurement Time	SOF	SkyDOAS			MeFTIR
			Alkanes	SO_2_	NO_2_	HCHO	CH_4_
Date	Weather Condition	Measurement Duration	N	N	N	N	N
3 November	Ave Temp: 9.7 °C Weather Condition: Sunny Ave Humidity: 22.2%	11.00 a.m.–01:00 p.m. (2 h)	4	4	4	4	4
4 November	Ave Temp: 9.7 °C Weather Condition: Sunny Ave Humidity: 31.50%	10.00 a.m.–12:00 p.m. (2 h)	4	4	4	4	4
5 November	Ave Temp: 9.7 °C Weather Condition: Sunny Ave Humidity: 37.49%	11.00 a.m.–02:00 p.m. (3 h)	4	6	6	6	4
8 November	Ave Temp: 9.7 °C Weather Condition: Sunny Ave Humidity: 27.31%	12.00 p.m.–02:00 p.m. (2 h)	4	4	4	4	4
9 November	Ave Temp: 9.7 °C Weather Condition: Sunny Ave Humidity: 15.88%	12.00 p.m.–03:00 p.m. (3 h)	5	5	5	5	4
10 November	Ave Temp: 9.7 °C Weather Condition: Sunny Ave Humidity: 32.83%	12.00 p.m.–02:00 p.m. (2 h)	4	4	4	4	4
11 November	Ave Temp: 9.7 °C Weather Condition: Sunny Ave Humidity: 34.36%	12.00 p.m.–03:00 p.m. (3 h)	6	4	4	4	4
13 November	Ave Temp: 9.7 °C Weather Condition: Sunny Ave Humidity: 34.59%	11.00 p.m.–01:00 p.m. (2 h)	5	4	4	4	4
16 November	Ave Temp: 9.7 °C Weather Condition: Cloudy Ave Humidity: 42.18%	12.00 p.m.–02:00 p.m. (2 h)	4	4	4	4	4
Total	-	21	40	39	39	39	36

**Table 5:** Daily wind direction and speed in zones S_1_ and S_2_.

Date	Zone S_1_				Zone S_2_			
	Wind Speed (m/s)		Wind Direction (Degree)		Wind Speed (m/s)		Wind Direction (Degree)	
	Average	Range (m/s)	Main Direction	Range (Degree)	Average	Range (m/s)	Main Direction	Range (Degree)
3 November	3.0 ± 0.4	2.8–3.0	NW	219–259	4.3 ± 0.2	3.7–4.9	NW	310–329
4 November	3.7 ± 0.3	3.3–4.0	NW	313–330	4.3 ± 0.1	4.1–4.9	NW	315–325
5 November	2.3 ± 0.3	1.9–2.4	SW	238–248	1.5 ± 0.5	1.5–3.1	SW	227–337
8 November	3.1 ± 0.4	2.8–3.6	SW	219–259	3.4 ± 1.0	3.9–5.7	NW	285–318
9 November	4.1 ± 0.3	3.8–4.4	NW	320–337	5.8 ± 0.6	5.2–6.7	NW	322–331
10 November	2.0 ± 0.4	1.6–2.6	NW	286–324	3.2 ± 0.6	6.6–8.4	NW	307–318
11 November	-	-	-	-	3.6 ± 0.4	4.0–4.3	NW	312–337
13 November	2.2 ± 0.7	1.5–3.9	NW	311–333	4.0 ± 0.8	3.9–5.3	NS	325–335
16 November	1.7 ± 0.2	1.6–2.0	EW	98–262	3.2 ± 0.6	2.2–4.2	NW	314–316

**Table 6:** Results of the PRTR survey vs. the measured values in zones S_1_ and S_2_.

	Zone S_1_	Zone S_2_
Measured values (kg/yr)	399,984	398,944
The PRTR survey result (kg/yr)	16,443	80,956

## Data Availability

No data have been used from elsewhere. And the data in this study is not available in any other places or websites.
